# P50, the Small Subunit of DNA Polymerase Delta, Is Required for Mediation of the Interaction of Polymerase Delta Subassemblies with PCNA

**DOI:** 10.1371/journal.pone.0027092

**Published:** 2011-11-02

**Authors:** Yujue Wang, Qian Zhang, Huiqing Chen, Xiao Li, Weijun Mai, Keping Chen, Sufang Zhang, Ernest Y. C. Lee, Marietta Y. W. T. Lee, Yajing Zhou

**Affiliations:** 1 Institute of Life Sciences, Jiangsu University, Zhenjiang, Jiangsu, People's Republic of China; 2 Department of Biochemistry and Molecular Biology, New York Medical College, Valhalla, New York, United States of America; Tulane University Health Sciences Center, United States of America

## Abstract

Mammalian DNA polymerase δ (pol δ), a four-subunit enzyme, plays a crucial and versatile role in DNA replication and various DNA repair processes. Its function as a chromosomal DNA polymerase is dependent on the association with proliferating cell nuclear antigen (PCNA) which functions as a molecular sliding clamp. All four of the pol δ subunits (p125, p50, p68, and p12) have been reported to bind to PCNA. However, the identity of the subunit of pol δ that directly interacts with PCNA and is therefore primarily responsible for the processivity of the enzyme still remains controversial. Previous model for the network of protein-protein interactions of the pol δ-PCNA complex showed that pol δ might be able to interact with a single molecule of PCNA homotrimer through its three subunits, p125, p68, and p12 in which the p50 was not included in. Here, we have confirmed that the small subunit p50 of human pol δ truthfully interacts with PCNA by the use of far-Western analysis, quantitative ELISA assay, and subcellular co-localization. P50 is required for mediation of the interaction between pol δ subassemblies and PCNA homotrimer. Thus, pol δ interacts with PCNA via its four subunits.

## Introduction

Chromosomal DNA replication in eukaryotes requires at least three major DNA replicative polymerases: α (pol α), δ (pol δ), and ε (pol ε). Current views of the eukaryotic replication indicate that pol α/primase synthesizes RNA primers plus short stretches of DNA to initiate Okazaki fragment synthesis, and the actual elongation of RNA-DNA primers is performed by pol δ (but not pol ε) in a process termed “polymerase switching” that involves replication factor C (RFC) and proliferating cell nuclear antigen (PCNA) [1], [2], [3], [4]. However, pol δ is also capable of performing leading strand synthesis [5], [6]. The roles of pol ε in replication are still not completely clear. Studies in budding yeast indicate that pol ε may function in mammalian DNA replication, synthesizing most of the DNA on the leading strand template [7], [8]. Although we do not know just how interchangeable the functions of pol δ and pol ε are at the replication fork *in vivo*, clearly both are involved in chromosomal replication. They may do so in roles that are specific or overlapping in the various processes involved in chromosomal replication besides replication of DNA, which range from replication initiation, sister chromatid cohesion, the nature of the chromatin that is encountered, and DNA damage checkpoint responses [5], [9], [10].

The subunit composition of pol δ has been controversial and may vary among different eukaryotes. In yeast, *S. pombe* pol δ consists of four subunits, Pol3, Cdc1, Cdc27 and Cdm1 [11], [12], [13]. While *S. cerevisiae* pol δ is a trimer of the first three subunits, Pol3p, Pol31p/Hys2, and Pol32p [14], [15]. The smallest non-essential subunit Cdm1 has no apparent homologue in budding yeast [13]. The mammalian pol δ was initially characterized as a two-subunit complex of 125 kDa and 50 kDa [16], [17], [18], [19]. The p125 catalytic subunit, containing both the polymerase and exonuclease catalytic domain, is homologous to fission yeast Pol3 and budding yeast Pol3p, whereas the small subunit p50 is a homologue of Cdc1 and Pol31p/Hys2. It was later shown that mammalian pol δ has two additional subunits, p68 and p12 [20], [21], [22], [23]. Thus, similar to *S. pombe* enzyme, the mammalian pol δ consists of at least four subunits, forming a heterotetrameric complex.

Maintaining high-fidelity chromosomal DNA replication is essential for the preservation of genomic integrity and avoidance of the mutations which can lead to disease. Mammalian cells respond to DNA damage by a host of defense mechanisms which include activation of cell cycle checkpoints and DNA repair mechanisms [24], [25]. In addition to its crucial role in DNA replication, pol δ also plays a significant role in DNA repair, and is generally regarded as the primary enzyme which performs re-synthesis (gap-filling) in various DNA repair processes [5], [24], [26]. The subunit composition of pol δ complex *in vivo* may vary with cellular response to different events. As replication stress or genotoxic agents trigger the degradation of the p12 subunit, pol δ is consequently converted from a heterotetramer (p125/p50/p68/p12) to a trimer (p125/p50/p68) lacking the p12 subunit [27]. This converted trimer has altered enzymatic properties with a less activity to perform translesion synthesis when it encounters DNA base lesions, a greater proofreading ability for the insertion of wrong nucleotides and extension of mismatched primers, and enhanced ability for the detection of errors in both primers and templates over its parent enzyme [28]. More recent studies indicate that this trimer is more likely to mediate single-nucleotide base excision repair (SN BER) in uracil-intiated BER *in vitro* [29]. These alterations of enzymatic property may be partly due to the loss of p12 subunit, resulting in a conformational change of pol δ, which alters its fidelity by modulating the rate and proofreading 3′ to 5′ exonuclease activity, a more rapid and frequent transfer of the DNA primer from the catalytic center to the exonuclease active center [30].

The function of pol δ as a chromosomal DNA polymerase is dependent on its association with PCNA which functions as a molecular sliding clamp here. PCNA also plays a crucial and versatile role in many DNA transactions where it acts as a scaffold for the recruitment and organization of protein complexes which involve DNA transactions both in DNA replication and repair. However, the identity of the subunit of pol δ which directly interacts with PCNA and is therefore primarily responsible for the processivity of the enzyme remains controversial. All four subunits of pol δ have been reported to contain a PCNA-binding motif and the interaction of each subunit with PCNA has been suggested to be primarily responsible for the high processivity of pol δ [20], [23], [31], [32], [33]. These observations are consistent with that the interactions of human pol δ with PCNA are likely to be multivalent, but leaves open the questions of which, or how many, of the subunits are involved in the interaction with PCNA during DNA replication or repair processes; whether multiple arrangements of pol δ and PCNA exist *in vivo*, and whether these may be involved in the different process in which both pol δ and PCNA play a role. Previously, we proposed a model for the network of protein-protein interactions of the pol δ-PCNA complex in which the pol δ might be able to interact with a single molecule of PCNA homotrimer through its three subunits, p125, p68, and p12 [31]. We had not been able to confirm the interaction of p50 with PCNA at that time because the weakest binding of the p50 to PCNA. In this work we have re-examined the interaction of p50 with PCNA using far-Western analysis, quantitative ELISA assay, and subcellular co-localization. Our results indicate that the small subunit p50 of human pol δ indeed interacts with PCNA. We have also modified the model of protein-protein interactions of the pol δ-PCNA complex to include p50 in which pol δ interacts with PCNA via its four subunits.

## Materials and Methods

### Reagents and chemicals

All reagents and chemicals used in this study were purchased from Sigma-Aldrich, Gibco-BRL, and Invitrogen except as otherwise indicated. A rabbit polyclonal antibody against p50 was prepared in this work. A mouse monoclonal antibody against PCNA (PC-10) was obtained from Santa Cruz Biotechnology.

### Production of polyclonal antibody

For immunogen preparation, the GST-tagged p50 was generated by the PCR using the plasmid pCDNA3.1(+)-FLAG containing a full-length p50 as a template [34]. The generated PCR fragments were digested with *Bam*HI and *Eco*RI, subcloned in-frame into the *Bam*HI-*Eco*RI sites of pGEX-5X-3, and sequenced. The primer pairs used here were as follows: Forward, 5′-CGCGGATCCGCATGTTTTCTGAGCAGGCTGCC-3′; Backward, 5′-CCGGAATTCTCAGGGGCCCAGCCCCAGGCC-3′ (the *Bam*HI and *Eco*RI sides are underlined, respectively). The generated clones were transformed into *E. coli* DH5-α cells.

GST-tagged p50, expressed in one liter of DH5-α cells harboring full-length p50 of human pol δ in vector pGEX-5X-3, was purified on glutathione-Sepharose 4B beads (Amersham Pharmacia Biotech). Non-tagged p50, used as antigen, was released by proteolysis with Factor Xa and the glutathione S-transferase was removed with glutathione-Sepharose. Released fractions of non-tagged p50 were combined and then purified by FPLC chromatography on Mono Q 5/50 GL column (GE Healthcare). The highly purified protein was analyzed by mass spectrometry to further confirm amino acid sequence before immunization.

The polyclonal antibody against small subunit p50 of human pol δ was produced by immunizing rabbits with highly purified non-tagged p50 protein. The collected antiserums were precipitated at 40-50% saturation of (NH_4_)_2_SO_4_ and followed by the low-salt purification on a column packed with 5-ml Pierce Protein A/G Plus Agarose as described [35].

### Mass spectrometry analysis

The specific bands corresponding to human pol δ small subunit p50 protein were excised manually from the polyacrylamide gel with a sterile scalpel and digested with trypsin according to Li's method [36]. The digested samples were analyzed by an ultraflex MALDI-TOF–TOF instrument (Bruker, Germany). Peptide mass fingerprinting (PMF) was performed by comparing the masses of identified peptides to NCBI protein database using the MASCOT search engine (http://www.matrixscience.com).

### Purification of His-tagged protein and human pol δ four-subunit complex

His-tagged p68 expressed in *E. coli* BL21DE3(plys) was purified by the use of nickel-nitrilotriacetic acidagarose (Qiagen) and further purified by ion exchange chromatography on FPLC Mono Q column (GE Healthcare) as described previously [31]. The protocol for rapid isolation of recombinant pol δ four-subunit complex generated from *Bombyx mori* bioreactor was as described [29].

### Digoxigenin (DIG)-labeling of PCNA

The recombinant human PCNA expressed in *E. coli* was purified to near homogeneity by a protocol as previously described [29], [37]. Highly purified PCNA was then labeled using the DIG Protein Labeling Kit (Roche Diagnostics) according to the manufacture's instructions.

### Far-Western blotting — Overlay Blotting with PCNA-DIG

PCNA-DIG overlay for the detection of the interaction between p50 and PCNA was performed as described previously by the use of DIG-labeled PCNA as a probe [38]. Increasing amounts of non-tagged p50 or purified pol δ4 complex from *Bombyx mori* bioreactor [29] were run on 12% SDS-PAGE and transferred to nitrocellulose membranes. The buffers used in the overlay experiment were purchased from the manufacturer (Roche Diagnostics). The membranes were blocked with 1x blocking solution in 1x Maleic acid buffer overnight at 4°C, washed for 10 minutes with 1x washing buffer, and incubated with DIG-labeled PCNA in PBS for one hour (1∶10,000 dilution of the 0.15 mg/ml PCNA-DIG stock). The membranes were washed with 3x30 minutes with washing buffer, incubated with Anti-DIG-HRP at 1∶10,000 dilutions in 100 mM Tris-HCl, pH 7.5/150 mM NaCl, followed by 3x20 minutes washing with washing buffer. The signals were detected using enhanced chemiluminescence lighting, ECL (Pierce) and the membranes were exposed to film.

### Enzyme-linked Immunosorbent Assay (ELISA) for detection of p50-PCNA protein-protein interactions

The ELISA for the protein-protein interactions was basically described as [31]. Briefly, purified non-tagged p50 and His-p68 (as a positive control) were diluted to a concentration of 0.001 µg/µl in carbonate buffer, pH 9.6. Each 100 µl of dilutions was added to the wells of a 96-well ELISA plate and allowed to coat overnight at 4°C. For the control experiment, BSA was substituted for subunits in the coating step. Wells were rinsed three times with ELISA buffer (50 mM Tris-HCl, pH 7.8, 150 mM NaCl, 0.5% Tween-20), then blocked with ELISA buffer containing 3% BSA by incubating plate for 2 hours at room temperature. Increasing amounts of PCNA in ELISA buffer containing 1% BSA were added to appropriate wells and allowed to be incubated at 4°C overnight. Wells were emptied and washed four times with ELISA buffer. The primary mouse monoclonal antibody against PCNA (PC-10) was diluted at 1∶2000 in ELISA buffer containing 1% BSA, added to each well, and incubated for one hour at room temperature. The plate was then washed four times with ELISA buffer. The secondary antibody of goat anti-mouse IgG-horseradish peroxidase (Pierce) was diluted at 1∶5,000 in ELISA buffer containing 1% BSA and added to appropriate wells to incubate for 30 minutes at room temperature. Wells were rinsed five times with ELISA buffer. The signals were detected using TMB substrate (Pierce) and then quenched with 1 N sulfuric acid for 10 minutes. Absorbance readings were taken at 450 nm. ELISA was also performed by coating a 96-well plate with 100 ng of purified recombinant human PCNA and adding increasing amounts of non-tagged p50, His-p68, or BSA. The rabbit polyclonal antibody against p50 or p68 [29] was used to detect bound p50 or His-p68 to PCNA, respectively.

### Western blot analysis to test the efficiency of obtained polyclonal antibody

A western blot protocol for testing the efficiency of obtained polyclonal antibody was as described previously [29]. An electrophoresis was performed by loading 100 µg each well of Hela cell total lysate onto 12% SDS-PAGE and separated proteins were transferred to a nitrocellulose membrane. The membrane was then stained with Ponceau S and cut into several pieces corresponding to each loading well. These pieces of membrane were blocked with 5% w/v nonfat dry milk in TBST buffer (20 mM TrisHCl, pH 7.4, 150 mM NaCl, 0.05% Tween 20) for one hour at room temperature. The slices were then incubated with decreasing amounts of antibody concentration for one hour at room temperature at 4°C. After three 15-min washes in TBST, the slices were incubated with AP-conjugated goat anti-rabbit IgG (Pierce) for one hour and washed with TBST 3 times for 10 min. Perfect Protein Western Blot Kit (Novagen) was used for signal generation.

### Immunocytochemistry

Immunocytochemistry was performed as described previously [34]. HeLa cells were grown in 4-well slides and fixed with 100% methanol and further permeabilized with 0.1% Triton X-100 in PBS. Endogenous p50 was immunostained with purified anti-p50 rabbit polyclonal antibody at optimized concentration in 2% BSA/PBS for one hour at room temperature and washed three times with PBS. Endogenous PCNA was immunostained with anti-PCNA mouse monoclonal antibody (PC-10) at 1∶ 200 dilutions, washed three times with PBS, and then incubated with Rhodamine-X-conjugated anti-rabbit IgG (Red) and Alexafluor488-conjugated (green) anti-mouse IgG (Jackson ImmunoResearch) at room temperature for one hour in the dark. DNA was counterstained with DAPI. Slides were viewed on an Axiovert 200 M fluorescence microscope (Zeiss) and analyzed with the Axiovision software (Zeiss).

### Protein concentration determination

Protein concentrations were determined by the Bradford method with bovine serum albumin (BSA) as a standard, or by “in-gel” determination using catalase as a protein standard [28].

## Results and Discussion

### Production of polyclonal antibody against human pol δ small subunit p50

**Figure 1:**
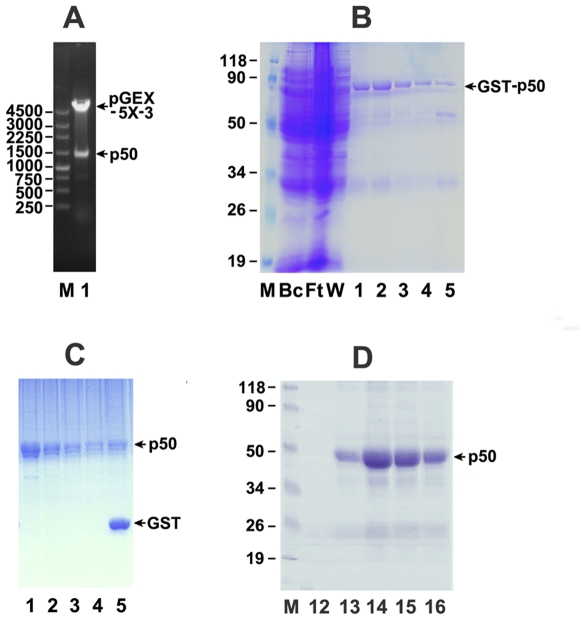
Analysis for the preparation of the immunogen for the production of polyclonal antibody against small subunit p50 of human DNA pol δ. **A:** Agarose gel electrophoresis. M, DNA marker in bp; Lane 1, a selected colony of pGEX-5X-3-p50 digested with *Bam*HI and *Eco*RI. **B:** Coomassie Blue stained SDS-PAGE analysis for the purification of GST-p50 protein on glutathione-Sepharose 4B column. The lysates (BC), flow-through (FT), and eluted fractions were analyzed on 12% SDS-PAGE followed by Coomassie Blue staining. Protein maker is indicated in lane M as kDa. The position of GST-p50 protein is indicated by an arrow. **C:** Coomassie Blue stained SDS-PAGE analysis for release of non-tagged p50 by Factor Xa. Lane 1-4: fractions of released p50; Lane 5: the beads containing GST and unreleased p50 after digestion reaction. **D:** Coomassie Blue stained SDS-PAGE analysis for the peak fractions of released p50 protein further purified on Mono Q column.

**Figure 2:**
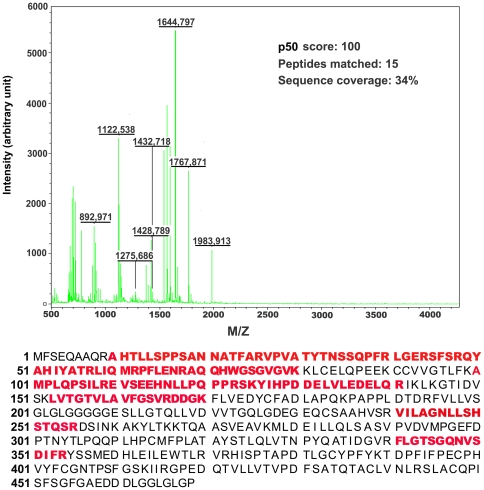
MALDI spectra of tryptic digestion of recombinant p50 subunit of human DNA pol δ. The identified protein, score, amino acid sequence coverage and the number of identified peptides are shown. The sequences of identified peptides shown in bold red covered 34% sequences against the deduced amino acid sequence of p50.

Our initial effort for polyclonal antibody production with purified His-tagged fusion p50 as antigen which was expressed in *E. Coli* cells harboring full-length p50 in pET-28b(+) vector was unsuccessful because of the lower sensitivity and specificity of the obtained antibody, which may due to the quality of the immunogen. The purified His-tagged fusion protein was not pure enough as immunogen for the purpose of immunization by the purification on nickel-nitrilotriacetic acidagarose beads and further on Mono Q 5/50 GL column only. Alternatively, we chose non-tagged p50 in its native form as immunogen by construction of full-length p50 fragment into pGEX-5X-3 vector (Figure 1A), expression of GST-tagged p50 in *E. Coli* and purification on glutathione-Sepharose 4B beads (Figure 1B), release of non-tagged p50 with Factor Xa protease from fusion proteins which are bound to Glutathione Sepharose 4B beads (Figure 1C), and further purification of released fractions of non-tagged p50 on FPLC Mono Q column (Figure 1D). About 4 mg of highly purified p50 protein from one liter of DH5-α cells was obtained. The amino acid sequence of obtained protein was further verified by mass spectrometry (Figure 2). Fifteen peptide fragments were identified with MOWSE score of 100 and matched against the deduced amino acid sequence of p50 with 34% sequence coverage.

**Figure 3:**
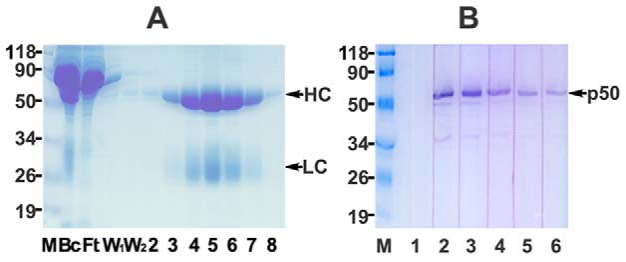
Analysis for the production of polyclonal antibody. **A:** Coomassie Blue stained SDS-PAGE analysis for the purification of polyclonal antibody against p50 after affinity chromatography on a 5-ml Protein A/G Plus column. The dialyzed sample in PBS after ammonium sulfate precipitation (Bc), flow-through (Ft), wash (W_1_ and W_2_), and eluted fractions were analyzed on 12% SDS-PAGE followed by Coomassie Blue staining. Protein marker in kDa is indicated by “M”. The heavy and light chains are marked by arrows. **B:** Measurement of sensitivity and specificity of purified antibody by Western blotting with Hela cell extracts. The six lanes show decreasing concentrations of antibody contained 0.8, 0.4, 0.2, 0.1, 0.05 (lane 2-6), and 0 µg/ml (lane 1) of antibody/slice membrane, respectively. The detected endogenous p50 is marked by an arrow.

After immunizing two New Zealand rabbits using our highly purified non-tagged p50 protein as immunogen, about 200 mg of polyclonal antibody was purified in high purity from about 120 ml of collected antiserum (final termination bleeds) by the precipitation of (NH_4_)_2_SO_4_ and further on a Protein A/G Plus Agarose packed column (Figure 3A). The efficiency of obtained antibody was tested by Western blotting using Hela cell extracts. Optimized concentration of purified antibody at 0.05–0.1 µg/ml could result in a high sensitivity for detecting the interaction between antibody and endogenous p50 subunit of human pol δ in good specificity (Figure 3B, lane 5 and 6).

### A direct interaction of p50 with PCNA was confirmed by far-Western blotting- PCNA-DIG overlay

**Figure 4:**
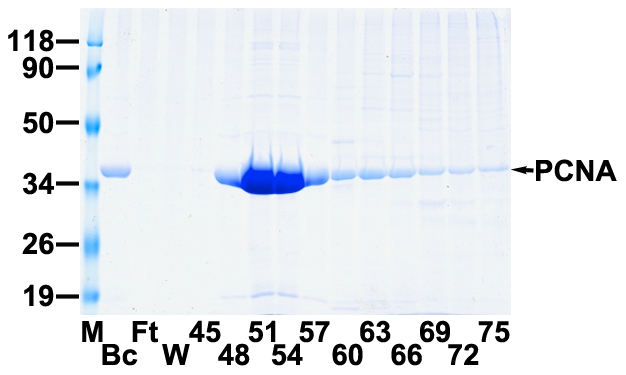
Coomassie Blue stained SDS-PAGE analysis for the purification of recombinant human PCNA after FPLC chromatography on Mono P column. Wild-type PCNA from the expression of one liter of DH5-α cells was purified on Q-sepharose column. Collected peak fractions were further purified on 4-ml Mono P HR 5/20 column. The starting material dialyzed in TGEED (Bc), flow-through (Ft), wash (W), and eluted fractions (45-75) were analyzed on 12% SDS-PAGE followed by Coomassie Blue staining. Protein marker is indicated in kDa by “M”. the position of PCNA is indicated by an arrow.

**Figure 5:**
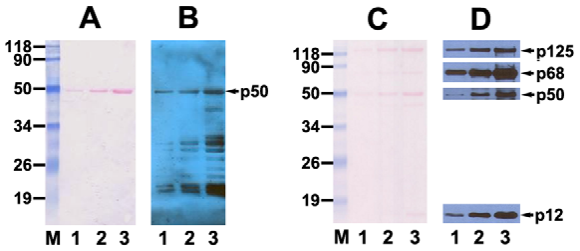
Far-Western analysis to detect the binding of PCNA to p50. Highly purified non-tagged p50 (Figure 6A, Lane 2) and pol δ4 [29] were run on 12% SDS-PAGE gel. Separated proteins were transferred onto nitrocellulose membrane and stained with Ponceau S. **A:** Lane 1-3 shows loaded 0.25, 0.5, 1 µg of p50. **C:** Lane 1-3 shows loaded 0.125, 0.25, 0.5 µg (based on p50) of recombinant pol δ4. The membranes were then subjected to PCNA-DIG overlay assay as described in “Materials and Methods”. The signals were detected by ECL and the membranes were exposed to the films. **B:** Exposure of the film for p50 which exactly corresponds to Ponceau S stained membrane of panel A. **D:** Exposure of the films for individual four subunits of pol δ, respectively, which exactly correspond to Ponceau S stained membrane of panel C. Exposure of the film for p50 in both panel B and panel D was slightly higher than those used for other subunits. Protein marker is indicated in kDa by “M”. Four subunits of pol δ are indicated by arrows.

Despite the fact that PCNA was first identified as a processivity factor for pol δ about 25 years ago [39], the identity of the subunit of pol δ that directly interacts with PCNA and is therefore primarily responsible for the processivity of the enzyme remains controversial. It seems that all four subunits of the pol δ interact with PCNA by the perusal of the literature mentioned in introduction. If p50 could indeed interact with PCNA [33], its binding should be a weakest one comparing with other subunits' that is probably why we could not confirm it before. Here, we applied a more sensitive approach to the testing of direct interaction between p50 and PCNA, far-Western analysis. The recombinant PCNA expressed in one liter of DH5-α cells harboring human PCNA in pTACTAC vector [29] was purified to near homogeneity by a Q-sepharose column and then further by FPLC chromatography on a 4-ml Mono P HR 5/20 column. The elution behavior of PCNA on Mono P was shown in Figure 4. The peak fraction 51 was used for the labeling of digoxigenin. The final DIG labeled PCNA solution was obtained with the concentration of 0.15 mg/ml. Highly purified non-tagged p50 and recombinant four-subunit complex of human DNA pol δ from silkworm bioreactor [29] were loaded at increasing amounts onto 12% SDS-PAGE and then transferred onto nitrocellulose membranes. The transferring efficiency of separated proteins onto membranes was judged by Ponceau S staining (Figure 5A and 5C). The membranes were then subjected to the PCNA overlay assay. As shown in Figure 5D, PCNA directly bound to the subunit p125, p68, and p12, which are in agreement with the previous reports. Surprisingly, p50 can also interact with PCNA which was judged by the bound PCNA both on the non-tagged p50 (Figure 5B) and on p50 subunit of pol δ complex (Figure 5D, the piece of film for p50). Although its binding is weaker than other subunits', the p50-PCNA interaction still could be clearly observed by a slight overexposure of the films which allow visualization of bound PCNA. This is the first time we confirmed the direct interaction of p50 with PCNA because of its weaker binding.

It should be noted that in our experimental condition, PCNA exhibited strong interaction with a number of truncated products, especially between 19 KDa and 34 KDa (Figure 5B), which is not visible by Ponceau staining (Figure 5A). Previous study revealed that a synthesized peptide fragment containing the N-terminal MRPFL sequence of p50, a homologue of sliding clamp binding motif of RB69 DNA polymerase (LFDMF) and T4 DNA polymerase (LDFLF), appeared strong competition with p50 for binding to PCNA [33]. Many bands other than p50's shown in our far-Western assay suggested that our purified non-tagged p50 from *E. coli* expression is not pure enough. It contained a lot of contaminants with MRPFL-like sequence which might compete with p50 for binding to PCNA, which could account for the bands between 19 KDa and 34 KDa in our far-Western assay. Strong interactions of PCNA with a dizzying variety of partners suggested that PCNA may have other, as yet unknown, functions besides its role as a “sliding clamp”, a mobile platform for the docking of an impressive array of enzymes responsible for the replication and repair of DNA.

### The interaction of p50 with PCNA was further verified by quantitative ELISA assays

**Figure 6:**
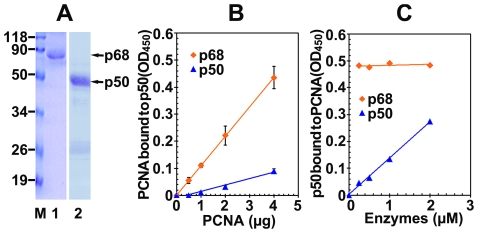
Quantitative ELISA assay to verify the interaction of p50 with PCNA. **A:** Coomassie blue stained SDS-PAGE analysis for the highly purified proteins used in ELISA assays. M, protein marker in kDa; Lane 1, highly purified His-p68. Lane 2, highly purified non-tagged p50. **B:** Interaction of p50 with PCNA by adding PCNA to coated p50. The assays were performed as described in “Materials and Methods”. The 96-well plates were coated with 100 ng of p50, His-p68 and BSA. ELISAs using increasing amounts of PCNA (horizontal axis) were then performed using an antibody PC-10 to detect the bound PCNA to p50. The p68 was taken as positive control while BSA was taken as “noise”. Absorbance readings were taken at 450 nm and the values were plotted after subtraction of the control values with BSA. Each assay was performed three times and the standard deviations are shown by the error bars. **C:** Interaction of p50 with PCNA by adding p50 to coated PCNA. The 96-well plate was pre-coated with 100 ng of PCNA. Increasing amounts of 50, His-68 (as a control), or BSA were then added (horizontal axis). Bound proteins to PCNA were recognized by an antibody against p50 or p68 to detect the interaction between PCNA and p50 or p68.

In order to further verify the interaction between p50 and PCNA, the quantitative ELISA assays were performed. The purified non-tagged p50 (Figure 6A, lane 2), His-p68 which was used as a positive control (Figure 6A, lane 1), and BSA which was used as a negative control were coated onto a 96-well ELISA plate, adjusting proteins to 100 ng per well. Increasing amounts of PCNA were then added to protein-coated wells. The bound PCNA was detected by mouse monoclonal antibody PC-10. As shown in Figure 6B, PCNA bound to the wells coated with p50 with a linear response following the increase of PCNA amounts from 0 to 4 µg per well. Absorbance readings at 450 nm indicated a relative weaker interaction of PCNA-p50, *i.e.*, 21% of that for PCNA-p68. Alternatively, a 96-well plate was coated with purified PCNA, and increasing amounts of non-tagged p50, His-p68, or BSA were then added. The bound p50 or His-p68 to PCNA was detected by the rabbit polyclonal antibodies against p50 (in this work) or anti-p68 [29]. Absorbance readings at 450 nm showed that p50 bound to PCNA-coated wells also as a linear response following the increase of p50 concentration from 0 to 2 µM while only small amount of His-p68, only 0.025 µM, brought interaction of p68-PCNA to a saturation level (Figure 6C). In such case, although two interaction levels could not be directly comparable because it depends on the sensitivity of antibodies in which two different antibodies were used to detect bound His-p68 and p50 to PCNA-coated wells. Nevertheless, these data proved that p50 indeed interacts directly with PCNA although this binding level is relatively weak.

### The intracellular localization of p50 and PCNA provides an evidence for a possible physical interaction between p50 and PCNA *in vivo*

Many proteins have been identified which interact with PCNA in which PCNA acts as an assembly platform for the replication machinery. The third subunit p68 was shown to be a nuclear protein which was excluded from the nucleolus and was localized to replication foci during S phase [40]. These foci or factories represent a conglomeration of proteins involved in DNA replication and post-replicative processing which are brought together at sites of DNA replication. This pattern of localization was shared with many proteins involved in DNA replication such as PCNA, pol α, Fen1, DNA ligase I, RFC, RPA etc [41], [42].

**Figure 7:**
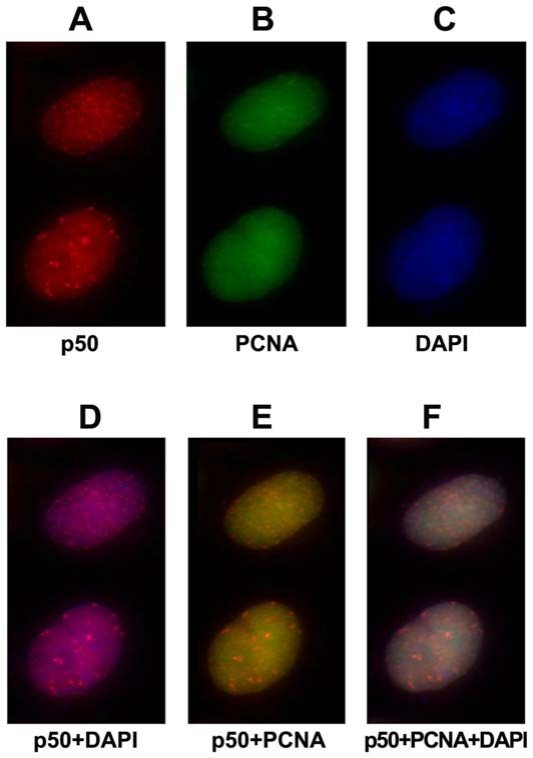
Co-nuclear staining of p50 and PCNA. Hela cells were fixed, permeabilized, and co-stained for p50 and PCNA using indirect immunofluorescence in which an anti-p50 rabbit polyclonal antibody was used for p50 while an anti-PCNA mouse monoclonal antibody PC-10 was used for PCNA. **A:** Rhodamine-X-conjugated anti-rabbit IgG secondary antibody was used for p50 (red). **B:** Alexafluor488-conjugated anti-mouse IgG secondary antibody was used for PCNA (green). **C:** DNA was counterstained with DAPI immunofluorescence (blue). **D:** Merger for p50 (panel A) and DAPI (panel C). **E:** Merger for p50 (panel A) and PCNA (panel B). **F:** Merger for p50 (panel A), PCNA (panel B), and DAPI (panel C). Cells were analyzed and photographed with an Axiovert 200 M fluorescence microscope (Zeiss).

To understand better the likely physiological interaction of p50 with PCNA *in vivo*, we investigated their intracellular localization in cultured cells. The cultured Hela cells were pre-extracted with 100% methanol, which would remove soluble PCNA, leaving only molecules involved in DNA replication. The anti-p50 polyclonal antibody used here specifically recognized one major band migrating with apparent molecular size slightly larger than 50 kDa at 0.05-0.1 µg/ml of the antibody concentration when total lysates of HeLa cells were immunoblotted (Figure 3B, lane 5 and 6). The subcellular co-localization of endogenous p50 and PCNA to the nuclear region (Figure 7C) by DAPI staining was clearly observed on microscopy which was in a pair-wise fashion by indirect immunofluorescence when the Hela cells were co-stained with anti-PCNA mouse monoclonal antibody and anti-p50 rabbit polyclonal antibody (Figure 7). The endogenous p50 localized to distinct nuclear spots which are typical of replication foci (Figure 7A). It looks likely that the observed subcellular localization of p50 is similar to the pattern of localization of p68 in U2OS cells [40], localized to DNA replication foci. This observation strongly supports a possible direct physical interaction between p50 and PCNA *in vivo*. However, the nature of which p68, or p50, or both is mainly required for mediation of the interaction between pol δ subcomplex(es) and PCNA during DNA replication *in vivo* is still needed to be further investigated in which the replication foci should be visualized more clearly by a proper approach, for example, pulse-labeling cells with BrdU and staining nuclei with a fluorescent antibody against BrdU after DNase treatment.

**Figure 8:**
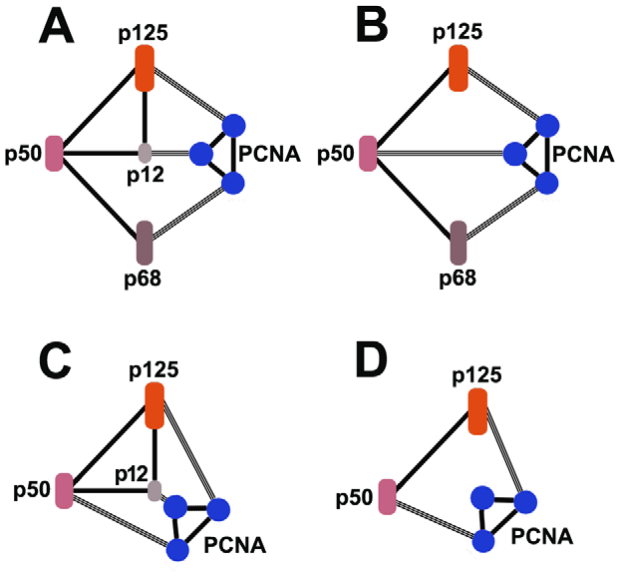
A model for the network of protein-protein interactions that may be involved in the assembly of the pol δ-PCNA complex. **A:** Complete structure. The inter-subunit protein-protein contacts for both pol δ and PCNA are shown as solid lines. Interactions between pol δ subunits and PCNA subunits are shown by the tripled lines. PCNA is shown as having the ability to act as a trivalent molecule. **B and C:** The protein-protein interaction network for the p125/p50/p68 trimer (panel B) and for the p125/p50/p12 trimer (panel C). In such models, p50 provides an alternative interaction with one monomer of PCNA homotrimer retaining three trivalent interactions of PCNA monomers. **D:** The protein-protein interaction network for 125/p50. Only two PCNA monomers retain trivalent interactions.

There is some evidence to support an assumption that there may exist multiple subassemblies of pol δ *in vivo*. Pol δ could be converted from heterotetramer to a heterotrimer through the degradation of the p12 subunit in cultured mammalian cells in which this heterotrimer could be isolated as an intact complex (p125/p50/p68) with altered properties [27], [28], [30]. Yet the heterotetrimer could be converted to a two-subunit complex as a core enzyme form of p125/p50 through the cleavage of p68 and p12 by human calpain-1 (unpublished data). Another phenomenon also suggested this assumption. The recombinant human pol δ could be assembled in a silkworm bioreactor as multiple complexes and these subassemblies could be isolated as dimer (p125/p50), trimer lacking p12, and four-subunit complex, respectively, in different eluted fractions on FPLC Mono Q chromatography [29]. Thus, an interconversion between the pol δ heterotetramer and trimer lacking p12, or even core dimer might occur *in vivo*, if not all, at least partially. We previously gave a proposed model for the interactions between pol δ subunits and PCNA based on the suggestion that these contacts take place with a single PCNA homotrimer [31]. Pol δ might be able to interact with a single molecule of PCNA homotrimer through its three subunits, p125, p68, and p12. The fact that the p50 interacts with PCNA and possible multi-arrangement of pol δ adds to the complexity of this issue. There remains an important question of how arrangements of pol δ and PCNA permit pol δ to adopt flexible configurations with PCNA in which its spatial orientation can be shifted between several modes and both pol δ and PCNA play a role in different DNA transactions. Here, we proposed a model of network for protein-protein interactions of pol δ subassemblies-PCNA complexes to include p50 in which pol δ interacts with PCNA via its four subunits as shown in Figure 8.

In this model, the interactions between pol δ and PCNA subunits are shown with the assumption in which these contacts take place with a single PCNA homotrimer. In the complete structure, six of the seven participants are each involved in three protein-protein interactions (Figure 8A). P50 is excluded from the interaction with PCNA because of its weaker binding to PCNA. In this case, whether the p50 subunit is involved in other DNA transactions and the nature of its role are still unknown. Removal of p12 or p68 converts the interaction network to one in which p50 provides an alternative interaction with one monomer of PCNA homotrimer, retaining three trivalent interactions of PCNA monomers (Figure 8B and 8C). While in the case of removal of both p12 and p68, the interaction network would convert to one in which only two PCNA monomers retain trivalent interactions (Figure 8D). This conversion results in one PCNA monomer free from the interaction with pol δ, which may permit a flexible configuration between pol δ and PCNA in which its spatial orientation can be shifted between several modes. Based on this model, we would argue that p50 may play an important role in converting pol δ and PCNA into an integrated assembly. We still could not get a conclusion of which, p50, or p68, or both, is mainly required to mediate the interaction between pol δ and PCNA. Mammalian p125 has been shown to bind to PCNA. However, the question of whether the p125 interaction with PCNA is also engaged in the pol δ-PCNA complex remains to be experimentally verified. Here, our prospective model may be incomplete or only partially correct but does provide a novel perspective of pol δ-PCNA interactions and p50 may be required to mediate the interaction of pol δ with PCNA.

In summary, we have confirmed that the p50 weakly binds to PCNA by far-Western analysis, quantitative ELISA assay, and subcellular localization. P50 may play a role in mediation of the interaction between pol δ subcomplexes and PCNA by the examination of the network of interactions in a proposed model of the PCNA-pol δ as an integrated structure.
